# Supplementary material for: SOLeNNoID: a deep learning pipeline for solenoid residue detection in protein structures
Source: Bioinformatics. 2025 Jul 21;41(8):btaf415. doi: 10.1093/bioinformatics/btaf415 (PMC12342502; doi:10.1093/bioinformatics/btaf415)
Supplement: btaf415_Supplementary_Data [file btaf415_supplementary_data.pdf]

SOLeNNoID: A Deep Learning Pipeline For Solenoid Residue Detection in Protein Structures Supplementary Material

Georgi I. Nikov, Daniella Pretorius and James W. Murray

July 13, 2025

1 Supplementary Methods

1.1 Training/validation dataset details

The training/validation dataset comprises the data from the REPETITA paper [Marsella et al., 2009], as well as additional beta-solenoid structures.

In the REPETITA paper, the authors take 32 solenoid proteins from a review of solenoid protein structures [Kobe and Kajava, 2000]. Then they use the TESE server [Sirocco and Tosatto, 2008] to add additional solenoid domains at a 35% pairwise sequence identity threshold to the original set of 32 structures. This yielded a final set of 105 solenoid structures. The TESE server was also used to obtain non-solenoid structures with different topologies and no detectable sequence similarity. The REPETITA dataset therefore already includes a measure of redundancy removal. This dataset has not been filtered beyond the work of the original authors. We added 12 further beta-solenoid structures to cover the range of beta-solenoid cross-sectional shapes. Optional sentence: Comparing the solenoid protein training and validation datasets using CD-HIT-2D [Fu et al., 2012] with a threshold of 40% sequence identity and a word size of 2 results in 17/22 validation set structures which pass this threshold.

Prediction Time Test

The prediction time test was conducted using the 6,040 AlphaFold2-predicted *S. cerevisiae* structures (v1 database) in the EBI AlphaFold database. The script was executed on the Imperial College HPC with 16 CPU cores, 96 GB RAM and 4 RTX6000 GPUs. The prediction time was defined from before loading a structure to after obtaining solenoid predictions for the structure.

Training Set				
	α-solenoid	α/β -solenoid	β-solenoid	Non-solenoid
Number of structures	26	13	41	196
Avg. number of residues	292	263	310	255
Avg. % of solenoid residues	78	78	60	0
Number of residues	6031	2735	7201	57988
Validation Set				
	α-solenoid	α/β-solenoid	β-solenoid	Non-solenoid
Number of structures	7	4	11	50
Avg. number of residues	346	246	327	269
Avg. % of solenoid residues	75	75	53	0
Number of residues	1880	756	1813	16016
Test Set				
	α-solenoid	α/β-solenoid	β-solenoid	Non-solenoid
Number of structures	11	6	15	49
Avg. number of residues	581	513	450	263
Avg. % of solenoid residues	74	78	55	0
Number of residues	4023	2428	3265	18833

Table S1: **Training, validation and test set statistics.**

Data Processing and Visualisation

Data analysis was carried out using the Pandas library [Mckinney, 2010] in Python3 [Rossum and Drake, 2009]. Data visualisation was carried out using the Seaborn [Waskom, 2021], matplotlib [Hunter, 2007], matplotlib-venn (https://github.com/konstantint/matplotlib-venn) and logomaker [Tareen and Kinney, 2020] libraries. Protein structure visualisation was carried out using the nglview library [Nguyen et al., 2018] in Python3.

Supplementary Results

Prediction Time Test

To evaluate the time taken by SOLeNNoID to process a structure and make predictions, a test was conducted on the 6,040 v1 AlphaFoldDB predicted protein structures for *S. cerevisiae*. The result in Fig. S3 below shows that prediction time had a second order polynomial relationship with respect to the length of a protein. However, predictions for structures around 2500 amino acids took less than a minute under the conditions used, whereas predictions for structures up to around 1200 amino acids took less than 10 seconds. This demonstrates that SOLeNNoID can rapidly produce predictions even for large structures. In comparison, the TAPO, PRIGSA2 and RepeatsDB-Lite servers can take on the order of minutes to produce a prediction for a structure over 1000 amino acids.

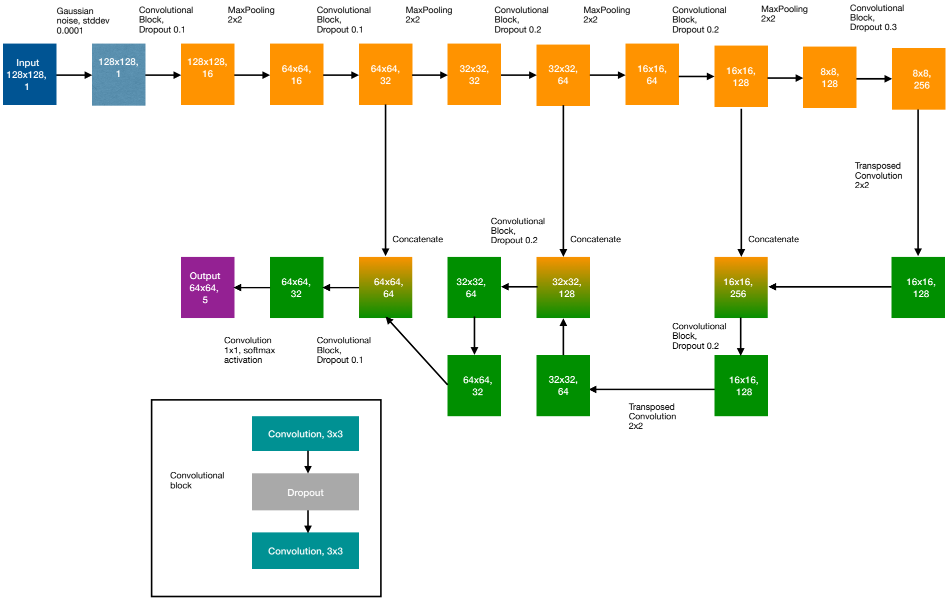

Figure S1: **Illustration of the U-Net architecture used in this work.** Each convolutional block consists of the three layers shown in the box. Each square denotes the dimensions of the input, intermediate tensor or output.

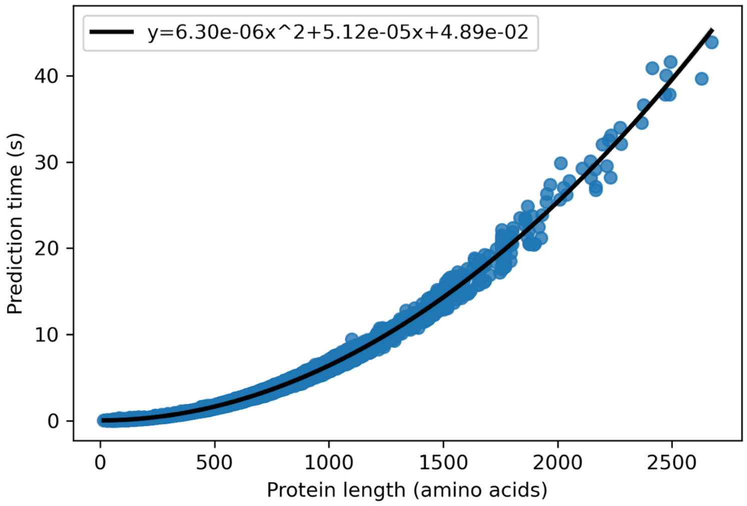

Figure S2: **Relationship between protein length and prediction time for SOLeNNoID method on the YEAST v1 AlphaFoldDB dataset.** Top left - second order polynomial equation fit to the data using numpy.polyfit.

	Precision			
Class	SOLeNNoID	TAPO	PRIGSA2	RDB-lite
Non-solenoid	**0.786**	0.780	0.509	0.494
Solenoid	0.870	0.855	**0.954**	0.892
	**Recall**			
Class	SOLeNNoID	TAPO	PRIGSA2	RDB-lite
Non-solenoid	0.760	0.729	**0.958**	0.892
Solenoid	**0.886**	**0.886**	0.488	0.494
	**F1-score**			
Class	SOLeNNoID	TAPO	PRIGSA2	RDB-lite
Non-solenoid	**0.773**	0.754	0.665	0.636
Solenoid	**0.877**	0.870	0.646	0.636
	**MCC**			
	SOLeNNoID	TAPO	PRIGSA2	RDB-lite
	**0.651**	0.625	0.455	0.387

Table S2: **Comparison of method performance on the solenoid test set as a binary (solenoid/non-solenoid) classification problem.** The metrics used are precision, recall and F1-score for single classes and multi-class MCC as a global metric. Values in bold represent the best performance across methods.

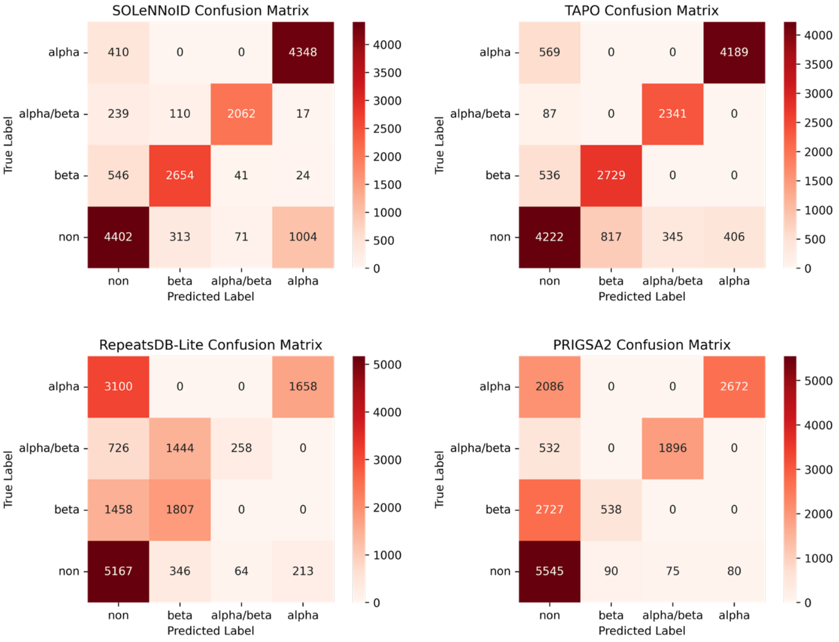

Figure S3: **Multi-class confusion matrices for SOLeNNoID, TAPO, PRIGSA2 and RepeatsDB-Lite on the solenoid test set.** True labels are shown on the y-axis and predicted labels are shown on the x-axis. The numbers represent the number of protein residues placed within each category of the confusion matrix. A darker red colour indicates a larger number of residues.

Class	Precision	Recall	F1-score
Non-solenoid	0.935	0.912	0.924
β-solenoid	0.860	0.813	0.836
α/β-solenoid	0.948	0.849	0.896
α-solenoid	0.770	0.914	0.835
MCC			
0.811			

Table S3: **Performance of SOLeNNoID on the whole test set as a multi-class classification problem.**

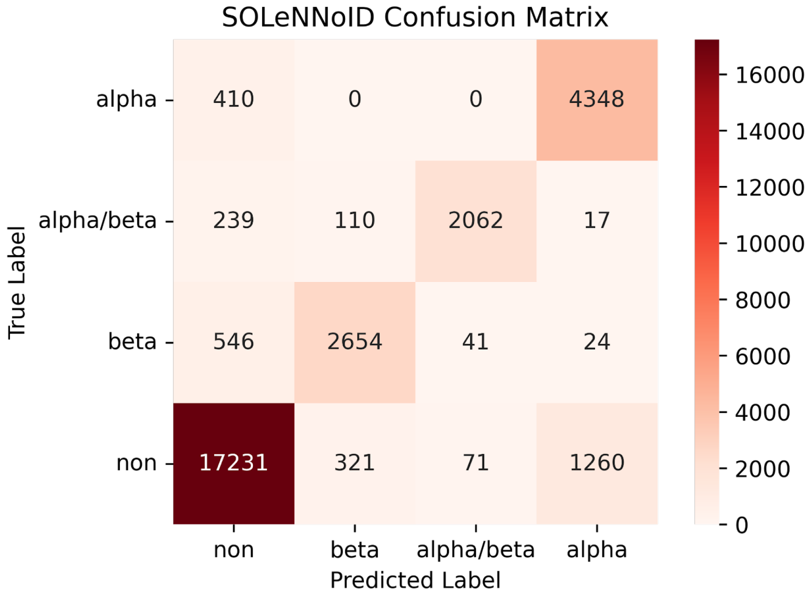

Figure S4: **Multi-class confusion matrix for the SOLeNNoID model on the full test dataset.** True labels are shown on the y-axis and predicted labels are shown on the x-axis. The numbers represent the number of protein residues placed within each category of the confusion matrix. A darker red colour indicates a larger number of residues.

	α-solenoid	α/β-solenoid	β-solenoid
Number of chains	7371	981	974
Number of PDB IDs	3326	490	366
Min. number of solenoid residues	36	49	28
Mean number of solenoid residues	348	273	198
Max. number of solenoid residues	3340	732	631

Table S4: **Statistics for PDB entries detected as solenoids by SOLeNNoID.**

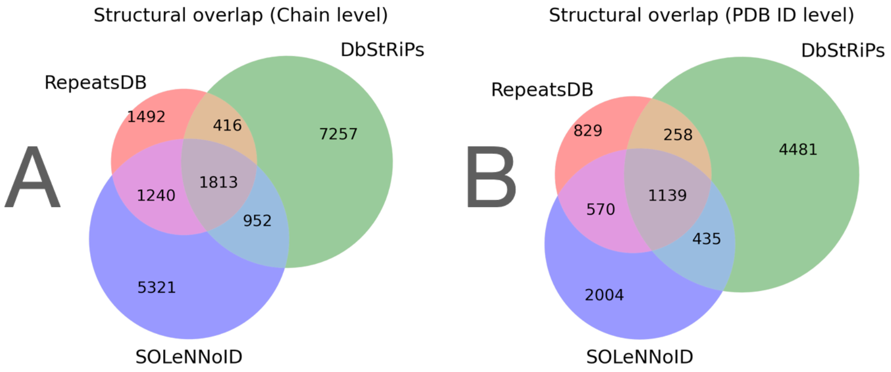

Figure S5: **Venn diagrams showing overlap between the structures detected as solenoid by SOLeNNoID and solenoid structures in the RepeatsDB and DbStRiPs databases.** Red - solenoid structures found only in the RepeatsDB database, green - solenoid structures found only in the DbStRiPs database, blue - solenoid structures found only using SOLeNNoID. Grey - solenoid structures found in all databases, pink - solenoid structures found both in RepeatsDB and by SOLeNNoID, cyan - solenoid structures found both in DbStRiPs and by SOLeNNoID, yellow - solenoid structures found both in RepeatsDB and DbStRiPs. A) Overlap between databases and SOLeNNoID predictions when considering unique individual chains. B) Overlap between databases and SOLeNNoID predictions when considering unique PDB IDs.

References

L. Fu, B. Niu, Z. Zhu, S. Wu, and W. Li. CD-HIT: accelerated for clustering the next-generation sequencing data. *Bioinformatics*, 28(23):3150–3152, Dec. 2012.

J. D. Hunter. Matplotlib: A 2D graphics environment. *Computing in Science and Engineering*, 9:99–104, 5 2007.

B. Kobe and A. V. Kajava. When protein folding is simplified to protein coiling: The continuum of solenoid protein structures. *Trends in Biochemical Sciences*, 25:509–515, 10 2000.

L. Marsella, F. Sirocco, A. Trovato, F. Seno, and S. C. Tosatto. REPETITA: Detection and discrimination of the periodicity of protein solenoid repeats by discrete Fourier transform. *Bioinformatics*, 25:289–295, 6 2009.

W. Mckinney. Data structures for statistical computing in python. 2010.

H. Nguyen, D. A. Case, and A. S. Rose. NGLview–interactive molecular graphics for Jupyter notebooks. *Bioinformatics*, 34:1241–1242, 4 2018.

G. V. Rossum and F. L. Drake. Python 3 reference manual; createspace. *Scotts Valley, CA*, page 242, 2009.

F. Sirocco and S. C. E. Tosatto. TESE: generating specific protein structure test set ensembles. *Bioinformatics*, 24(22):2632–2633, Nov. 2008.

A. Tareen and J. B. Kinney. Logomaker: beautiful sequence logos in python. *Bioinformatics*, 36:2272–2274, 4 2020.

M. L. Waskom. seaborn: statistical data visualization. *Journal of Open Source Software*, 6:3021, 4 2021.
